# Association between Internet use and depression among older adults in China: the chain-mediating role of volunteer activity participation and friend network

**DOI:** 10.3389/fpubh.2024.1403255

**Published:** 2024-09-24

**Authors:** Yujiang Liu, Fang Li, Jian Sun

**Affiliations:** ^1^College of Public Administration, Nanjing Agricultural University, Nanjing, China; ^2^Jin Shanbao Institute for Agriculture and Rural Development, Nanjing Agricultural University, Nanjing, China

**Keywords:** Internet use, depression, Chinese older adults, friend network, volunteer activity participation

## Abstract

**Background:**

Depression is a significant burden on individuals and society, which requires our attention and action. As the aging wave collides with digitalization, further research is needed to understand how internet use relates to depression in older adults. This study aimed to investigate whether volunteer activity participation and friend networks played a chain mediating role in this relationship from the perspective of the socioemotional selectivity theory.

**Method:**

This study’s data was derived from the 2018 China Longitudinal Aging Social Survey (CLASS), comprising 5,558 samples. The study employed the OLS model for benchmark regression and multiple robust check methods, including altering variable settings and utilizing the instrumental variable model. In addition, the generalized structural equation model (GSEM) was used to analyze the mechanism.

**Result:**

Using the internet was significantly associated with reduced depression in older adults (coefficient = −0.9321, *p* < 0.001). The instrumental variable model confirmed this reduction (coefficient = −0.9200, *p* < 0.001). Moreover, we found that there were three indirect pathways of Internet use on depression among older adults: the mediating role of volunteer activity participation (all *p*-values <0.001), the mediating role of friend network (all *p*-values <0.001), and the chain mediating role of both factors (all *p*-values <0.001).

**Conclusion:**

Based on the research findings, we suggest mobilizing tech-savvy older adults to assist others in adopting digital technology and using the internet. We also suggest that the government could assist in creating older adult-friendly communities. Social workers could collaborate with tech-savvy older adults to organize various voluntary activities, encouraging more senior citizens to participate. In addition, we recommend that the community should consider the form of activities to help older adults make friends first rather than solely focusing on voluntary activities.

## 1 Introduction

Depressive disorder, ranking 12th in global disease burden and among the top three causes of Years Lived with Disability (YLDs) (1), has a global prevalence of 3.8%, with 5.0% among adults and 5.7% among those over 60 years old (2). China, as a moderately aging society with an accelerating aging process (3), faces an increasing need to address geriatric depression. As of June 2023, 13.0% of Chinese internet users are aged 60 and above (4). This growing digital engagement among older adults presents new opportunities for mental health interventions.

Numerous studies have explored the link between Internet use and depression among older adults. However, there is potential for further investigation, including mechanism analysis and variable selection. In terms of mechanism analysis, on the one hand, several empirical studies have delved into various aspects of Internet use, focusing specifically on usage purpose (5, 6), frequency (7), and skill (8) instead of whether older adults use the Internet. Most of them have found that chatting with family and friends online (6), using the internet more frequently (7), and improving internet skills (8) are all linked to improved depression levels in older adults. Despite these advances, there remains a critical need for more in-depth investigation into the underlying mechanisms that link Internet use with depression among older adults. On the other hand, several studies have investigated specific mechanisms such as interpersonal relationships (9), social networks (10), and social participation (11). However, these analyses have not thoroughly explored the interactions and mutual influences among these different factors. It’s important to consider the combined effects of multiple mechanisms, such as social participation and social networks (12), to better understand the complexity of how Internet use is associated with depression among older adults. In terms of variable selection, the concept of social participation is multifaceted and lacks a widely acknowledged definition (13). Some studies categorize social participation into three or four groups based on participation content, such as political, leisure, and voluntary participation (14, 15). Social networks can also be categorized into two main types: family networks and friend networks (16). Varied measurements may influence the analysis outcomes.

In this study, we select volunteer activity participation and friend networks as mediators to explore their potential role in the association between Internet use and depression among older adults. Friendship is a crucial element of social relations in later life, yet it has not been as extensively researched as other aspects (17). As people age, their inclination to cultivate new friendships through volunteering diminishes, but their motivation to seek value and maintain existing friendships in volunteering increases (18). While taking part in volunteer activities may help older adults meet others, changes in their motivation to volunteer could reduce their desire to cultivate new friendships. Additionally, as volunteer projects end or change, it can be difficult to maintain the relationships formed during the volunteering experience. Moreover, dedicating time and energy to volunteering may limit older adults’ interactions with their current friends. Therefore, it is essential to further explore the correlation between volunteer activity participation and friend networks. To our knowledge, this study is the first attempt to analyze the role of volunteer activity participation and friend networks as mediators in the relationship between Internet use and older adults’ depression, especially exploring their chain mediating role. By exploring the complex correlations among them, this research aims to provide comprehensive empirical evidence to formulate policies for improving depression among older adults, especially by focusing on the coordination of older adults’ volunteer participation and their friend networks.

## 2 Literature review and hypothesis development

### 2.1 Internet use and older adults’ depression

Existing research generally suggests that Internet use is linked to lower levels of depression (19, 20). Utilizing the internet for communication offers older adults a new means to connect with their families and peers, thereby helping them stay in touch and reducing their depression (21, 22). Internet use also empowers senior citizens to participate in more social activities, which can reduce depression (11). Furthermore, online social participation can maintain offline social participation in later life, which can mitigate the detrimental effect of pain on depression (23). However, excessive use of the Internet may lead to depression (24) and exacerbate social comparisons related to status or income, negatively impacting individual well-being (25).

### 2.2 The mediating role of friend networks

According to the socioemotional selectivity theory, as individuals age, they may perceive time limitations, leading them to prioritize present-oriented goals such as emotion regulation (26). Older people tend to choose familiar social partners more frequently to conserve emotional resources in the face of limited future opportunities (27). They can adjust their social networks to maintain stable intimate relationships (28), regardless of their cultural background or understanding of intimate partners (29, 30). Online social networks undergo similar changes, as older individuals usually have smaller online social network sizes but contain a higher proportion of actual friends (31, 32). Thus, regardless of whether older adults use the Internet or not, they tend to seek higher emotional experiences, such as maintaining close relationships. The Internet is a valuable tool that can help older adults quickly and efficiently share diverse information with their intimate partners, such as friends, using text, audio, video, etc. It is unnecessary for older adults to depend on direct, physical contact in order to sustain existing relationships. For example, an older adult may maintain a connection with an old friend who lives at a distance, even engaging in each other’s daily lives through instant video calls on a regular basis. This frequent and high-quality interaction helps keep their social relationships strong, preventing them from fading or breaking due to time and distance. In contrast, older individuals who do not use the Internet may experience situations that can be described as having the desire to maintain friendships but lacking the ability to do so effectively. They may only be able to stay in touch through occasional phone calls or letters, lacking frequent and meaningful interactions. As a result, those who use the internet are able to maintain and strengthen existing relationships, allowing them to sustain a relatively broader and deeper network of friends.

Social networks play a crucial role in the mental health of older adults. When their social networks diminish, they are more likely to experience feelings of loneliness and depression (33). Friend networks, as a component of social networks, are important for the mental health of older adults. More frequent social interactions with friends correlate with fewer depressive symptoms (34) while having fewer close friends is related to more depressive symptoms (35).

### 2.3 The mediating role of volunteer activity participation

The perception of limited time in the future may also prompt older adults to focus more on a sense of belonging and the meaning of life in order to gain emotional experience (36). Volunteering helps individuals achieve a sense of mattering and contributing (37, 38). The Internet is a highly efficient mobilization tool that can facilitate connections with potential volunteers (39). Consequently, they are more likely to participate in unpaid work for most voluntary organizations, particularly older adults (40). These older adults tend to engage more in social participation (41), community involvement (42), and volunteering (43).

Social participation can alleviate depression in older adults, including those who are widowed (44) or caregivers (45). More specifically, when older adults have higher levels of neighborhood social participation or community volunteer activities, they experience lower levels of depression (46–48).

### 2.4 Chain mediating role of volunteer activity participation and friend networks

Besides adjusting social networks and focusing on the meaning of life, as individuals age, they tend to selectively invest their time in activities that hold greater subjective meaning, while neglecting those that are less meaningful to them (49). Volunteering may not be as prioritized as spending time with family and close friends (50). Thus, age is found to be associated with higher social (maintaining existing friendships) and value (volunteering to express or act on important values) motives for volunteering, even after accounting for demographic characteristics and volunteering experience (51, 52). This may not motivate older individuals to cultivate new friendships through volunteering.

Volunteering among older adults can also lead to role conflicts (53). For example, caring for sick or disabled adults is a demanding task that can easily cause role conflicts (54). Moreover, older adults may not participate in volunteer activities with their peers and friends, especially in informal community volunteering. Therefore, further analysis is necessary to expand our understanding of this issue, especially concerning whether volunteer activity participation negatively correlates with the friend networks of older adults.

### 2.5 Research hypotheses

**Figure 1:**
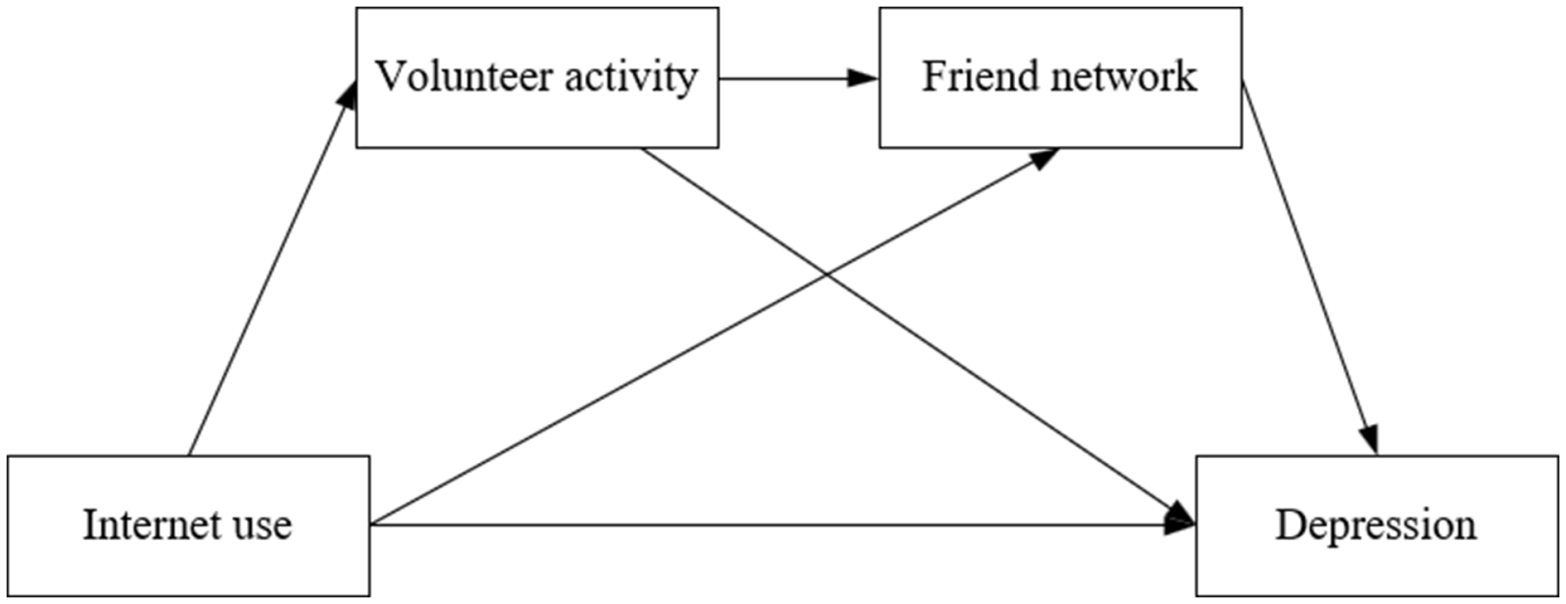
The analytical framework of the relationships among Internet use, volunteer activity, friend network, and older adults’ depression.

To sum up, to explore the relationship between Internet use and older adults’ depression, we construct a chain mediating model based on the socioemotional selectivity theory, as shown in Figure 1. This theory suggests that older adults prioritize present-oriented goals due to their perception of limited time in the future. As a result, they may adjust their social networks to maintain intimate relationships, participate in volunteer activities to find meaning in life, and selectively invest their time to gain more emotional experience (26, 27, 36). These behaviors have been found to be associated with lower levels of depression.

The Internet is an efficient tool that helps older adults overcome time and space constraints as they adjust their social networks and participate in volunteer activities. Older adults can easily maintain close relationships by staying connected and sharing more information through the Internet. However, older adults who do not use the Internet may find it challenging to maintain close relationships due to limitations of time and space, despite their willingness. They can also use the internet to find information about volunteer services quickly or to communicate more easily with the people involved in community volunteer programs. As a result, the Internet helps older adults efficiently allocate their time, focusing on meaningful activities and enhancing their emotional experience with intimate relationships and volunteer work.

However, changes in volunteer motivations and potential role conflicts may lead older adults involved in informal community volunteering to have fewer friend networks. The motivations for older adult individuals to participate in volunteer services may not primarily be to make new friends but rather to maintain existing friendships or pursue specific values. Additionally, older adults may not participate in some informal community volunteer work with their friends. Furthermore, while providing volunteer services, older adults may experience role conflicts. Therefore, these factors may be associated with a diminish in older adults’ friend networks. After considering the analysis presented, this study proposes the following hypotheses:

*H1*: Internet use is related to a decrease in depression among older adults.

*H2*: Internet use is associated with an increase in older adults’ friend networks.

*H3*: Older adults’ friend networks are linked to a decrease in depression among older adults.

H4: Friend networks play a mediating role in the relationship between Internet use and older adults’ depression.

*H5*: Internet use correlates with increased volunteer activity participation among older adults.

*H6*: Participation in volunteer activity is connected to lower depression in older adults.

*H7*: Volunteer activity participation plays a mediating role in the relationship between Internet use and older adults’ depression.

*H8*: Volunteer activity participation is negatively related to older adults’ friend networks.

*H9*: Volunteer activity participation and friend networks play a chain mediating role in the relationship between Internet use and older adults’ depression.

## 3 Method

### 3.1 Data and study sample

The empirical analysis data were sourced from the China Longitudinal Aging Social Survey (CLASS), a national longitudinal social survey conducted by the Renmin University of China, designed to investigate and analyze older adults’ basic situation. The respondents of this survey were Chinese older adults aged 60 and above.

Our study used data from CLASS2018, which comprised 11,418 individuals. The exclusion criteria were as follows: (1) individuals aged below 65 years; (2) Samples with ambiguous information (e.g., unable to provide a response, unknown, refusal to answer, etc.); (3) older adults with annual incomes over one million yuan; (4) Samples that did not fit the survey’s questions; (5) Samples that did not utilize the Internet for text, voice, or video communication; and (6) missing values for each variable. After preprocessing missing values and outliers, we obtained 5,558 valid samples. Empirical analyses were conducted using Stata (version 17.0).

### 3.2 Variable measurement

#### 3.2.1 Depression

The 9-item Epidemiologic Studies-Depression (CES-D) scale was used to measure depression (55). There were two items indicating negative feelings (lonely and upset), feelings of marginalization (having nothing to do and feeling useless), and somatic symptoms (having trouble sleeping and poor appetite), respectively. Furthermore, three items represented positive feelings: pleasure, enjoying life, and happiness. Each item was assigned a value of 0 (rarely or none of the time), 1 (some of the time), or 2 (most of the time). These ratings reflected the participant’s weekly frequency of experiencing each item. After reversing the coding of positively oriented items, we added up the scores of the nine items to obtain a depressive symptom score that ranged from 0 to 18. A higher score indicated a higher level of depression (Cronbach’s *α* = 0.7247).

#### 3.2.2 Internet use

The questionnaire included a question asking whether the respondent used the Internet, including various devices such as mobile phones. The values 1 to 5 indicated the frequency of Internet usage, ranging from daily to never using the Internet. We assigned a value of 0 to those who never used the Internet and 1 to those who did. Moreover, as previously mentioned in the exclusion criteria, this study did not include samples from individuals who did not utilize the Internet for text, voice, or video communication. Thus, in this paper, Internet use by older adults was defined as accessing the Internet through various devices and engaging in at least text, voice, or video communication.

#### 3.2.3 Voluntary activity participation

The questionnaire used to measure voluntary activity participation consisted of seven items: patrolling in the community, caring for other children and older adults, protecting the environment, tackling neighborhood disputes, accompanying chat, volunteering with required professional skills, and educating the next generation except their grandchildren. If respondents participated in any of them, they were assigned a score of 1. If they did not participate in any voluntary activities, they were given a score of 0.

#### 3.2.4 Friend network

Regarding evaluating older adults’ friend networks, CLASS 2018 utilized the Lubben Social Network Scale (LSNS-6) to gather data on family and friend networks. To evaluate friend networks, participants were asked three questions: (a) How many friends do you see or communicate with monthly? (b) How many friends can you share private matters without hesitation? (c) How many friends can give you a hand when you need it? These questions were designed to assess the breadth and depth of friend networks among the older adults. For example, the readiness to share personal matters without hesitation indicated a high degree of intimacy in the friendship, while the willingness to provide help when needed reflected the closeness and reliability of these friendships. Responses were rated on a scale of 0 to 5, with the options being none, one, two, three to four, five to eight, and nine or more. The sum of these questions’ scores determined older adults’ friend network scores. A higher score indicated that an older adult had both a greater number of connections and more intimate and supportive relationships within their friend network (Cronbach’s *α* = 0.8342).

#### 3.2.5 Control variables

This study selected control variables based on previous studies and data accessibility, including gender (male = 1, female = 0), age (years), education (illiterate = 1, old-style private school = 2, primary school = 3, secondary school = 4, high school = 5, college degree or above = 6), marital status (married = 1, widowed, divorced, and unmarried = 0), residency area (urban areas = 1, rural areas = 0), self-rated health (with 1–5 indicating very healthy to very unhealthy, respectively), activity of daily living (disability in activities of daily living, yes = 1, no = 0), chronic (the number of chronic diseases), smoke (have smoke behavior = 1, otherwise = 0), household income (logarithmically transformed), elder abuse (yes = 1, no = 0), and work status (with 1–5 indicating work every day, at least once per week, at least once each month, a couple of times a year, and never, respectively).

Furthermore, attitude toward aging was divided into general attitude toward aging and self-attitude toward aging. The general attitude toward aging was measured by the question, “Do you agree that current social changes are increasingly detrimental to the older adult?”, rated on a scale of 1–5, with 1 being strongly disagree and 5 being strongly agree. Similarly, there were seven questions used to measure self-attitude toward aging, including four positive questions (If I get the chance, I want to help the village/neighborhood committee with some tasks; I often feel the need to do more to help society; I now enjoy studying; and I think I’m still helpful to the society) and three negative questions (It’s hard to keep up with the fast-paced social changes; I struggle to accept many viewpoints nowadays; and new social policies are making it hard for me to accept). The process of assigning values was similar to the general attitude toward aging. After reversing the coding of positive questions, we added up the scores to obtain a self-attitude toward aging score (Cronbach’s *α* = 0.8227). The variable “negative event” was defined as the number of negative events older adults had experienced in the past 12 months.

### 3.3 Empirical model

Considering depression in older adults as a continuous variable, we used a linear regression as the benchmark model, expressed in Equation 1. The specific model setting was as follows:

(1) $$Depression_{i}=\alpha_{0}+\beta_{0}internet\_use_{i}+\sum \beta_{m}X_{mi}+\varepsilon_{i}$$

where $Depression_{i}$ denoted the depression of the i-th older adult, $internet\_use_{i}$ meaned whether the i-th older adult uses the Internet, $X_{mi}$ indicated other control variables, $\varepsilon_{i}$ represented the random error term, and $\beta_{0}$ was the coefficient of the independent variable.

Given the potential endogeneity issues, we used an instrumental variable model in the robustness check section. Equations 2 and 3 respectively represent the first and second stages in the two-stage least squares method. The model was set as follows:

(2) $$Internet\_use_{i}=\alpha_{0}+\beta_{0}network\_signal_{i}+\sum \beta_{m}X_{mi}+\varepsilon_{i}$$

(3) $$Depression_{i}=\alpha_{1}+\beta_{1}\bar{internet\_use_{i}}+\sum \beta_{m}X_{mi}+\varepsilon_{i}$$

Here, $network\_signal_{i}$ was the instrumental variable, $\bar{internet\_use_{i}}$ was the fitted value of Equation 2, $X_{mi}$ indicated other control variables, $\varepsilon_{i}$ represented the random error term, and $\beta_{1}$ was the coefficient of the independent variable.

Generalized structural equation modeling (GSEM) was used in this study to examine the mechanism underlying Internet use and older adults’ depression, which allowed for continuous, binary, and multinomial modeling (56). Stata 17’s “gsem” command was used to explore the specific mechanisms. Due to no goodness-of-fit indices from the GSEM procedure, we only reported the Bayesian information criterion (BIC) and Akaike Information Criterion (AIC) values for models (57).

## 4 Results

### 4.1 Descriptive analysis

Table 1 presents the descriptive statistics for all variables. It includes the mean and standard deviation (in parentheses) for continuous variables and frequencies for categorical variables like gender and residency area. A T-test was employed to analyze continuous variables, while the chi-square test was used for categorical variables. The average depression level among older adults was 6.7702. Specifically, Internet users within this age group exhibited an average depression level of 5.2828, compared to 7.0317 for non-Internet users. These findings suggested that Internet users exhibited lower levels of depression compared to non-Internet users (*T* = 15.845, *p* < 0.001). However, further research is needed to ascertain whether Internet use is negatively related to older adults’ depression.

**Table 1:** Descriptive statistics of variables, CLASS 2018.

Variable	Total (*n* = 5,558)	No internet use (*n* = 4,727)	Internet use (*n* = 831)	*χ*^2^ or *t*-test	*p*-value
Dependent variable					
Depression	6.7702 (2.9997)	7.0317 (2.9053)	5.2828 (3.0946)	15.845	*p* < 0.00
Independent variable					
Internet use	0.1495 (0.3566)				
Mediating variable					
Friend network	6.4003 (3.0714)	6.2511 (3.0737)	7.2491 (2.9180)	−8.696	*p* < 0.001
Volunteer activity participation				
No	3,796	3,317	479	51.2475	*p* < 0.001
Yes	1762	1,410	352		
Instrumental variable					
Network signal					
No signal	3,403	3,366	37	1.3e+03	*p* < 0.001
Have signal	2,155	1,361	794		
Control variable					
Gender					
Female	2,717	2,328	389	1.6811	*p* = 0.195
Male	2,841	2,399	442		
Age	73.0599 (6.5512)	73.7121 (6.6381)	69.3502 (4.4991)	18.220	*p* < 0.001
Education	2.9000 (1.3262)	2.7197 (1.2934)	3.9254 (1.0068)	−25.546	*p* < 0.001
Marital status					
No spouse	1828	1,676	152	94.3390	*p* < 0.001
Have a spouse	3,730	3,051	679		
Residency area					
Rural	2,184	2091	93	323.5119	*p* < 0.001
Urban	3,374	2,636	738		
Self-rated Health	2.6571 (0.8318)	2.6738 (0.8388)	2.5620 (0.7843)	3.578	*p* < 0.001
Activity of daily living					
No	4,851	4,100	751	8.4218	*p* = 0.004
Yes	707	627	80		
Chronic	1.5846 (1.5291)	1.5515 (1.5005)	1.7726 (1.6712)	−3.848	*p* < 0.001
Smoke					
No	3,792	3,259	533	7.5265	*p* = 0.006
Yes	1766	1,468	298		
Logged Household Income	8.2056 (1.4482)	8.0823 (1.4914)	8.9070 (0.8937)	−15.460	*p* < 0.001
Elder abuse					
No	5,256	4,474	782	0.4075	*p* = 0.523
Yes	302	253	49		
Work status	4.3782 (1.2861)	4.3275 (1.3205)	4.6667 (1.0230)	−7.042	*p* < 0.001
General attitude toward aging	2.6898 (1.1002)	2.7144 (1.0981)	2.5499 (1.1029)	3.979	*p* < 0.001
Self-attitude toward aging	20.9351 (3.5131)	21.2139 (3.3588)	19.3490 (3.9287)	14.371	*p* < 0.001
Negative event	0.2008 (0.4693)	0.1961 (0.4612)	0.2274 (0.5125)	−1.775	*p* = 0.076

Standard deviations are presented in parentheses; the categorical variable indicates the frequency of each type.

Regarding the independent and mediating variables, approximately 15% of older adults were Internet users. The analysis revealed that older adults who used the Internet had more friends (*T* = −8.696, *p* < 0.001) and were more likely to participate in volunteer activities (*χ*^2^ = −51.2475, *p* < 0.001). This study will further analyze and validate the proposed hypothesis by examining the relationship between these variables.

Regarding other control variables, rather than non-Internet users among older adults, silver surfers (Internet users among older adults) were likelier to be younger (*T* = 18.220, *p* < 0.001), had higher levels of education (*T* = −25.546, *p* < 0.001), resided in urban areas (*χ*^2^ = 323.5119, *p* < 0.001), possessed higher household incomes (*T* = −15.460, *p* < 0.001), and had fewer work frequency (*T* = −7.042, *p* < 0.001). The two groups had distinctions among different variables in health status and behavior. Explicitly speaking, silver surfers demonstrated better self-rated health (*T* = 3.578, *p* < 0.001) and activities of daily living situations (*χ*^2^ = 8.4218, *p* = 0.004). In contrast, a lower number of chronic diseases (*T* = −3.848, *p* < 0.001) and proportions in smoking behavior (*χ*^2^ = 7.5265, *p* = 0.006) were found in non-Internet users among older adults. Nonetheless, Internet users among older adults maintained a relatively optimistic attitude toward aging, both in general attitude (*T* = 3.979, *p* < 0.001) and self-attitude toward aging (*T* = 14.371, *p* < 0.001).

### 4.2 Correlation analysis

We used three different methods based on the types of variables. First, we applied Pearson correlation analysis for the correlation between continuous variables. Second, for the correlation between categorical variables and continuous variables, we used point-biserial correlation analysis because the categorical variables in this study were binary. The results of this analysis were bolded in the table; third, we assessed the correlation between categorical variables using Cramér’s V coefficient and conducted the chi-square test. Unlike the Phi coefficient, Cramér’s V coefficient produces consistent results for 2×2 contingency tables, and it can also be applied to larger contingency tables. The results were highlighted in both bold and italics.

Table 2 presents the results of the correlation analysis. The results indicated that internet use, voluntary activity participation, and the friend network were all significantly and negatively correlated with depression, with *p* < 0.001. Additionally, internet use and voluntary activity participation, as well as internet use and the friend network, were both significantly and positively correlated, with *p* < 0.001. Furthermore, voluntary activity participation and the friend network were negatively correlated, with *p* < 0.01. These findings provided initial support for the hypotheses outlined in the article, although further empirical analysis was necessary.

**Table 2:** Results of correlation analysis.

Variable	1	2	3	4	5	6
1. Depression	1.000					
**2. Internet use**	−0.208^***^	1.000				
**3. Volunteer activity participation**	−0.131^***^	0.096^***^	1.000			
4. Friend network	−0.142^***^	0.116^***^	−0.041^**^	1.000		
**5. Gender**	−0.044^**^	***0.017***	***0.002***	**0.008**	1.000	
6. Age	0.113^***^	−0.237^***^	**−0.024**	−0.080^***^	−0.031^*^	1.000
7. Education	−0.143^***^	0.324^***^	0.055^***^	0.113^***^	0.165^***^	−0.179^***^
**8. Marital status**	−0.149^***^	0.130^***^	0.046^***^	0.070^***^	0.255^***^	−0.309^***^
**9. Residency area**	−0.127^***^	0.241^***^	0.146^***^	0.044^**^	−0.054^***^	**0.014**
10. Self-rated Health	0.255^***^	−0.048^***^	−0.053^***^	−0.066^***^	−0.045^***^	0.114^***^
**11. Activity of daily living**	0.100^***^	−0.039^**^	***0.003***	−0.028^*^	−0.065^***^	0.194^***^
12. Chronic	0.136^***^	0.052^***^	−0.122^***^	0.024	−0.092^***^	0.095^***^
**13. Smoke**	**0.025**	0.037^**^	−0.133^***^	0.047^***^	0.419^***^	−0.032^*^
14. Logged Household Income	−0.163^***^	0.203^***^	0.183^***^	0.112^***^	0.034^*^	0.035^**^
**15. Elder abuse**	0.091^***^	***0.009***	0.040^**^	**0.005**	***0.003***	**0.002**
16. Work status	−0.059^***^	0.094^***^	0.099^***^	−0.010	−0.071^***^	0.154^***^
17. General attitude toward aging	0.252^***^	−0.053^***^	−0.098^***^	−0.035^**^	**0.011**	−0.007
18. Self-attitude toward aging	0.287^***^	−0.189^***^	−0.070^***^	−0.044^***^	−0.054^***^	0.104^***^
19. Negative event	0.153^***^	**0.024**	**−0.023**	0.015	**−0.023**	0.035^**^

Variable	7	8	9	10	11	12
7. Education	1.000					
**8. Marital status**	0.194^***^	1.000				
**9. Residency area**	0.319^***^	0.037^**^	1.000			
10. Self-rated Health	−0.056^***^	−0.089^***^	**−0.018**	1.000		
**11. Activity of daily living**	−0.049^***^	−0.082^***^	***0.022***	0.238^***^	1.000	
12. Chronic	0.008	−0.078^***^	0.063^***^	0.249^***^	0.165^***^	1.000
**13. Smoke**	0.108^***^	0.135^***^	−0.044^***^	**−0.012**	−0.077^***^	−0.052^***^
14. Logged Household Income	0.339^***^	0.114^***^	0.450^***^	−0.046^***^	**0.012**	0.023
**15. Elder abuse**	0.030^*^	***0.002***	***−0.014***	0.042^**^	0.149^***^	**0.009**
16. Work status	0.132^***^	−0.054^***^	0.373^***^	0.051^***^	0.078^***^	0.015
17. General attitude toward aging	0.030*	**−0.013**	**−0.025**	0.109^***^	**0.013**	0.034^*^
18. Self-attitude toward aging	−0.159^***^	−0.109^***^	−0.080^***^	0.140^***^	0.087^***^	0.044^**^
19. Negative event	0.005	−0.038^**^	**0.012**	0.114^***^	0.132^***^	0.149^***^

Variable	13	14	15	16	17	18	19
**13. Smoke**	1.000						
14. Logged Household Income	0.109^***^	1.000					
**15. Elder abuse**	***0.026***	**0.015**	1.000				
16. Work status	−0.080^***^	0.203^***^	**0.008**	1.000			
17. General attitude toward aging	0.075^***^	0.015	0.029^*^	−0.025	1.000		
18. Self-attitude toward aging	−0.047^***^	−0.142^***^	0.078^***^	0.032^*^	0.241^***^	1.000	
19. Negative event	0.039^**^	0.009	0.158^***^	0.037^**^	0.106^***^	0.056^***^	1.000

^***^p < 0.001, ^**^p < 0.01, ^*^p < 0.05; the bolded variables are binary categorical variables. The meaning of the Italic values is Cramér’s V coefficient.

### 4.3 VIF test

Before conducting regression analysis, it was vital to test for multicollinearity among variables in the regression using the VIF test. Table 3 presents the VIF test results, indicating that all variables have VIF values between 1.04 and 1.50, with an average of 1.21. Even the highest VIF value was only 1.50, lower than the standard of 10 set by the VIF test. This suggested that there was no serious multicollinearity problem in this study.

**Table 3:** VIF test results.

Variable	VIF	1/VIF
Internet use	1.24	0.805126
Friend network	1.04	0.960085
Volunteer activity participation	1.11	0.899408
Gender	1.32	0.756706
Age	1.27	0.786539
Education	1.36	0.737461
Marital status	1.22	0.821087
Residency area	1.50	0.667773
Self-rated Health	1.15	0.867691
Activity of daily living	1.14	0.873373
Chronic	1.14	0.878557
Smoke	1.29	0.774246
Logged Household Income	1.43	0.699618
Elder abuse	1.06	0.946910
Work status	1.22	0.819119
General attitude toward aging	1.10	0.905909
Self-attitude toward aging	1.16	0.864544
Negative event	1.08	0.929674

### 4.4 The association between internet use and older adults’ depression

Upon adding various control variables from model 1 to model 3, the independent variable’s significance level remained stable, implying that Internet usage had a negative relation with older adults’ depression regardless of some changes in coefficient values (coefficient = −0.9321, *p* < 0.001).

Regarding other variables, a negative association was found between older adults’ depression and both a friend network (coefficient = −0.0946, *p* < 0.001) and participation in community volunteer activities (coefficient = −0.3248, *p* < 0.001). This implied that having a greater degree of friend networks and engagement in volunteer activities was related to a decrease in older adults’ depression. Moreover, older adults with spouses were significantly correlated with lower depression levels than those without partners (coefficient = −0.4358, *p* < 0.001).

Regarding health variables, the benchmark regression revealed that self-rated health and older adults’ depression had a positive relationship (coefficient = 0.5719, *p* < 0.001). Likewise, chronic illness numbers had an identical association with depression among older adults (coefficient = 0.1337, *p* < 0.001). This suggested that as the number of chronic illnesses increased, so did the level of depression. Furthermore, smoking, as a health behavior, was linked to older adults’ depression. Compared to non-smokers, smokers were likelier to have higher levels of depression (coefficient = 0.2985, *p* < 0.001).

Socio-economic factors had a notable association with depression among older adults. A rise in household income was highly linked to older adults’ depression reduction (coefficient = −0.1485, *p* < 0.001). This relation was also applicable to work status. The frequency of paid job work was inversely related to depression levels in older adults (coefficient = −0.0919, *p* < 0.01). Furthermore, taking into account the experiences and attitudes of older adults toward aging was crucial, which could contribute to analyzing their association with older adults’ depression. Regression analysis showed that elder abuse was a strongly related event to older adults’ depression (coefficient = 0.7382, *p* < 0.001). Additionally, as the number of negative events increased, so did the levels of depression in this group (coefficient = 0.5648, *p* < 0.001). Furthermore, both general and self-attitudes toward aging had a positive linkage with depression among older adults. When the score for general attitude toward aging rose, there was a significant corresponding elevation in older adults’ depression (coefficient = 0.4464, *p* < 0.001). Similarly, self-attitude toward aging also became a significant factor in older adults’ depression (coefficient = 0.1385, *p* < 0.001) (Table 4).

**Table 4:** Regression results of the association between Internet use and older adults’ depression.

Variable	Model 1	Model 2	Model 3
Internet use	−1.7489^***^ (0.1153)	−1.5300^***^ (0.1144)	−0.9321^***^ (0.1091)
Friend network		−0.1232^***^ (0.0133)	−0.0946^***^ (0.0123)
Volunteer activity participation		−0.7664^***^ (0.0846)	−0.3248^***^ (0.0823)
Gender			−0.1106 (0.0817)
Age			0.0087 (0.0063)
Education			−0.0360 (0.0320)
Marital status			−0.4358^***^ (0.0828)
Residency area			−0.1134 (0.0871)
Self-rated Health			0.5719^***^ (0.0476)
Activity of daily living			0.0432 (0.1174)
Chronic			0.1337^***^ (0.0258)
Smoke			0.2985^***^ (0.0843)
Logged Household Income			−0.1485^***^ (0.0296)
Elder abuse			0.7382^***^ (0.1472)
Work status			−0.0919^**^ (0.0303)
General attitude toward aging			0.4464^***^ (0.0347)
Self-attitude toward aging			0.1385^***^ (0.0113)
Negative event			0.5648^***^ (0.0786)
Constant	7.0317^***^ (0.0423)	8.0302^***^ (0.1015)	3.0367^***^ (0.5679)
Number of observations	5,558	5,558	5,558
F-test	229.98^***^	124.56^***^	92.41^***^
R-squared	0.043	0.071	0.233

Robust standard errors are presented in parentheses. ^***^p < 0.001, ^**^p < 0.01, ^*^p < 0.05.

### 4.5 Robustness checks

Three methods were employed to test the robustness of the study, including changing the setting of the independent variable, changing how to measure the dependent variable, and using different models for analysis.

Firstly, we conducted robustness testing by altering the settings of the independent variable. In the benchmark regression, the measurement method for Internet usage was binary—whether or not older adults use the Internet. We replaced it with the Internet use frequency and recoded it. The values 1 to 5 indicated the frequency of Internet usage, with 1 representing never using the Internet, 2 representing a couple of times a year, 3 representing at least once per month, 4 representing at least once each week, and 5 representing daily use. The higher the value, the more frequent Internet usage.

Table 5 presents the outcome of changing the independent variables setting without altering other control variables. The correlation between Internet use and older adults’ depression remained stable (coefficient = −0.2690, *p* < 0.001), which coincided with the benchmark regression. The significance levels of other control variables also unchanged, such as friend network (coefficient = −0.0935, *p* < 0.001), volunteer activity participation (coefficient = −0.3260, *p* < 0.001), marital status (coefficient = −0.4353, *p* < 0.001), self-rated health (coefficient = 0.5707, *p* < 0.001), number of chronic diseases (coefficient = 0.1353, *p* < 0.001), smoking behavior (coefficient = 0.2947, *p* < 0.001), household income (coefficient = −0.1508, *p* < 0.001), etc. Thus, the results of this research could be considered robust.

**Table 5:** Robustness checks by employing a continuous variable to measure the Internet use.

Variable	Model 1	Model 2	Model 3
Internet use	−0.4942^***^ (0.0305)	−0.4356^***^ (0.0303)	−0.2690^***^ (0.0295)
Friend network		−0.1210^***^ (0.0133)	−0.0935^***^ (0.0123)
Volunteer activity participation		−0.7638^***^ (0.0844)	−0.3260^***^ (0.0821)
Gender			−0.1069 (0.0816)
Age			0.0076 (0.0063)
Education			−0.0324 (0.0320)
Marital status			−0.4353^***^ (0.0827)
Residency area			−0.1018 (0.0873)
Self-rated Health			0.5707^***^ (0.0475)
Activity of daily living			0.0383 (0.1173)
Chronic diseases			0.1353^***^ (0.0256)
Smoking			0.2947^***^ (0.0842)
Logged household income			−0.1508^***^ (0.0296)
Elder abuse			0.7411^***^ (0.1473)
Work status			−0.0893^**^ (0.0303)
General attitude toward aging			0.4427^***^ (0.0347)
Self-attitude toward aging			0.1373^***^ (0.0113)
Negative event			0.5656^***^ (0.0785)
Constant	7.5341^***^ (0.0604)	8.4599^***^ (0.1079)	3.4150^***^ (0.5757)
Number of observations	5,558	5,558	5,558
F-test	262.49^***^	135.31^***^	93.66^***^
R-squared	0.048	0.075	0.234

Robust standard errors are presented in parentheses. ^***^p < 0.001, ^**^p < 0.01, ^*^p < 0.05.

Secondly, we changed the way we measure the dependent variable. CES-D scale containing 12 items was applied to measure depression among older adults. Compared to the previous measurement, three new items were added to the CES-D scale, including “Did you feel like you did not have anyone to accompany you in the past week?,” “Have you felt overlooked in the past week?,” and “Have you felt isolated in the past week?.” Each item was coded as 1 = Never, 2 = Sometimes, and 3 = Often. We recoded their assigned values from 1 to 3 to 0 to 2 and then added the scores. Finally, depression ranged from 0 to 24 (Cronbach’s *α* = 0.7515).

Table 6 shows the analysis results after adjusting the dependent variable setting. Notably, including additional control variables did not alter the direction or significance level of the independent variable’s coefficient (coefficient = −0.9760, *p* < 0.001).

**Table 6:** Robustness checks by altering the method of dependent variable setting.

Variable	Model 1	Model 2	Model 3
Internet use	−2.0703^***^ (0.1544)	−1.7561^***^ (0.1532)	−0.9760^***^ (0.1468)
Friend network		−0.1672^***^ (0.0181)	−0.1309^***^ (0.0165)
Volunteer activity participation		−1.1907^***^ (0.1111)	−0.5440^***^ (0.1100)
Gender			−0.1461 (0.1121)
Age			0.0248^**^ (0.0086)
Education			−0.0267 (0.0429)
Marital status			−0.6118^***^ (0.1113)
Residency area			−0.0431 (0.1206)
Self-rated Health			0.7648^***^ (0.0652)
Activity of daily living			−0.2268 (0.1551)
Chronic			0.1895^***^ (0.0347)
Smoke			0.5173^***^ (0.1196)
Logged Household Income			−0.1965^***^ (0.0399)
Elder abuse			1.0464^***^ (0.1993)
Work status			−0.2267^***^ (0.0427)
General attitude toward aging			0.7688^***^ (0.0466)
Self-attitude toward aging			0.1358^***^ (0.0149)
Negative event			0.7535^***^ (0.1091)
Constant	11.5383^***^ (0.0582)	12.9429^***^ (0.1395)	6.1912^***^ (0.7655)
Number of observations	5,302	5,302	5,302
F-test	179.71^***^	117.41^***^	94.28^***^
R-squared	0.035	0.069	0.239

Robust standard errors are presented in parentheses. ^***^p < 0.001, ^**^p < 0.01, ^*^p < 0.05.

Thirdly, considering potential endogeneity issues, we applied the instrumental variable method for robustness analysis. In terms of selecting instrumental variables, we used whether there is a network signal in the house as the instrument variable. The reason was that Internet use is closely related to network signals, but there is unlikely a direct connection between network signals and the occurrence of older adults’ depression. The assignment method for network signal variables was as follows: If there is a network signal in the house, it is assigned a value of 1. Conversely, if there is no network signal, it is given a value of 0.

Table 7 presents the analysis results. The first-stage regression outcomes were displayed in Model 1, while Model 2 showed the second-stage regression outcomes. Model 1 indicated a significant positive coefficient of the network signal (coefficient = 0.3122, *p* < 0.001), with a highly significant F-test value (*F*-test = 988.69, *p* < 0.001), suggesting a correlation between the network signal and Internet use. Meanwhile, the weak identification test results were also needed to verify the effectiveness of the instrument variable. Regarding the Stock-Yogo weak IV test, the critical value of it at the 10% level is 16.38, much lower than the F-test value. This indicated that the network signal is not an invalid instrument variable. Moreover, there was no need for an over-identification test since the number of instrumental and possible endogenous variables is 1.

**Table 7:** Robustness checks by using instrumental variable analysis.

Variable	Model 1	Model 2
Network signal	0.3122^***^ (0.0099)	
Internet use		−0.9200^***^ (0.2683)
Friend network	0.0011 (0.0013)	−0.0947^***^ (0.0125)
Volunteer activities	0.0492^***^ (0.0093)	−0.3254^***^ (0.0828)
Gender	−0.0083 (0.0090)	−0.1104 (0.0818)
Age	−0.0084^***^ (0.0006)	0.0089 (0.0068)
Education	0.0435^***^ (0.0032)	−0.0367 (0.0344)
Marital status	0.0221^**^ (0.0085)	−0.4359^***^ (0.0827)
Residency area	0.0628^***^ (0.0085)	−0.1144 (0.0898)
Self-rated Health	0.0009 (0.0050)	0.5719^***^ (0.0475)
Activity of daily living	−0.0101 (0.0120)	0.0432 (0.1171)
Chronic	0.0155^***^ (0.0030)	0.1335^***^ (0.0263)
Smoke	0.0007 (0.0097)	0.2983^***^ (0.0844)
Logged Household Income	−0.0231^***^ (0.0030)	−0.1486^***^ (0.0298)
Elder abuse	0.0081 (0.0188)	0.7381^***^ (0.1469)
Work status	0.0081^**^ (0.0029)	−0.0920^**^ (0.0303)
General attitude toward aging	−0.0073^*^ (0.0036)	0.4465^***^ (0.0347)
Self-attitude toward aging	−0.0108^***^ (0.0012)	0.1387^***^ (0.0116)
Negative event	0.0106 (0.0097)	0.5645^***^ (0.0786)
Constant	0.8190^***^ (0.0565)	3.0274^***^ (0.5942)
Number of observations	5,558	5,558
F-test	988.69^***^	
R-squared	0.3385	
Wald chi-squared		1575.90^***^
Stock-Yogo weak IV test critical values (10%)		16.38
Endogeneity test p-value		0.9606

Robust standard errors are presented in parentheses. ^***^p < 0.001, ^**^p < 0.01, ^*^p < 0.05.

Based on the results of Model 2, it could be seen that the result was consistent with the benchmark regression and other robust checks, indicating that the result of the study is robust. In addition, the endogeneity test yielded a *p*-value of 0.9606, suggesting that the study is not affected by endogeneity issues.

### 4.6 Mechanism analysis

**Figure 2:**
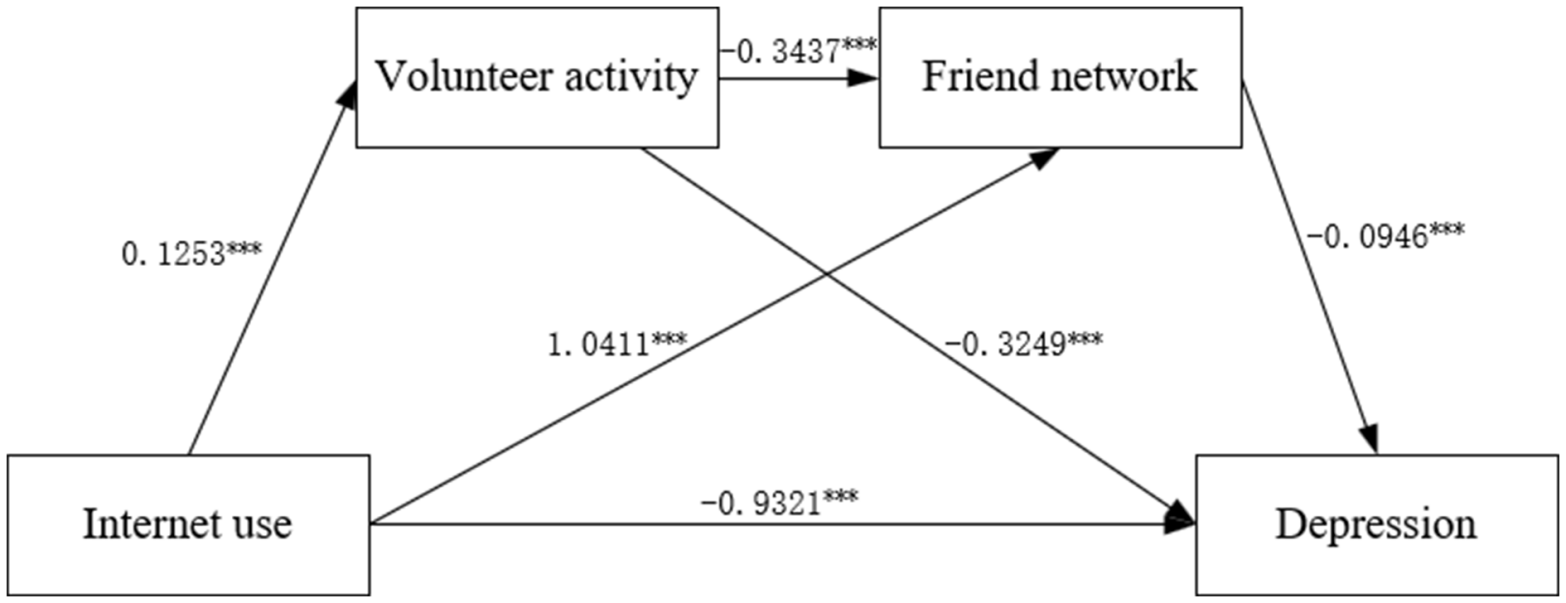
Generalized structural equation model results of the relationship among Internet use, volunteer activity participation, friend network, and older adults’ depression. ^***^p < 0.001, ^**^p < 0.01, ^*^p < 0.05. The standard error is the robust standard error. The control variables in this model are identical to the previous models. Log pseudolikelihood = −30,941.167, Log Likelihood = −30,941.17, Degree of Freedom = 27, Akaike Information Criterion (AIC) = 61,936.33, Bayesian information criterion (BIC) = 62,115.15.

We intended to investigate the previously proposed mechanism in this section. Figure 2 clearly depicts the generalized structural equation model results and shows that Internet use and friend networks among older adults have a positive relation (coefficient = 1.0411, *p* < 0.001). Additionally, the friend network was negatively related to older adults’ depression (coefficient = −0.0946, *p* < 0.001). Therefore, the friend network was a mediator in this path, which validates Hypotheses 1, 2, and 3. Similarly, we found a positive relationship between Internet use and participation in volunteer activities (coefficient = 0.1253, *p* < 0.001). Furthermore, participation in volunteer activities was negatively associated with depression in older adults (coefficient = −0.3249, *p* < 0.001). These results validated the role of volunteer activity participation, thereby verifying Hypotheses 4, 5, and 6. Moreover, a noteworthy inverse relationship existed between engaging in volunteer work and the size of the friend network (coefficient = −0.3437, *p* < 0.001). Consequently, volunteer activity participation and friend networks also formed a chain mediating role in the analytical framework, which validates Hypotheses 7 and 8.

### 4.7 Further analysis

After analyzing the relationship between Internet use, volunteer activity participation, friend networks, and older adults’ depression, we wanted to discuss the participation intensity instead of solely considering whether older adults participate in community volunteer activities. The same items applied to measure volunteer participation were also used to measure activity participation intensity. However, the method of variable setting changed. Each item had response options ranging from “never” to “almost every day,” rated on a scale of 0 to 4. We calculated an activity intensity score by counting each item’s score. The total score ranged from 0 to 28, indicating higher participation intensity (Cronbach’s *α* = 0.8839).

**Figure 3:**
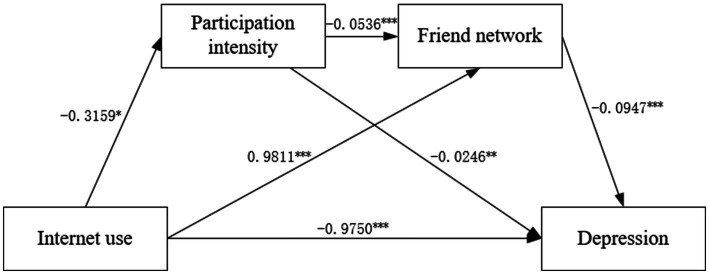
Generalized structural equation model results of the relationship among Internet use, participation intensity, friend network, and older adults’ depression. ^***^p < 0.001, ^**^p < 0.01, ^*^p < 0.05. The standard error is the robust standard error. The control variables in this model are identical to the previous models. Log pseudolikelihood = −43401.925, Log Likelihood = −43401.93, Degree of Freedom = 27, Akaike Information Criterion (AIC) = 86857.85, Bayesian information criterion (BIC) = 87036.67.

Figure 3 depicts the results of the generalized structural equation model. We observed a negative correlation between Internet usage and the intensity of activity participation (coefficient = −0.3159, *p* < 0.05). This contradicted the prior model. The intensity of participating in activities was negatively correlated with depression among older adults (coefficient = −0.0246, *p* < 0.01). The direction and significance of the path of Internet usage, the friend network, and older adults’ depression were consistent with those shown in Figure 2. This meaned that Internet use and the friend network had a positive relation (coefficient = 0.9811, *p* < 0.001), and the friend network was negatively linked to older adults’ depression (coefficient = −0.0947, *p* < 0.001). In addition, participation intensity was still negatively correlated with the friend network (coefficient = −0.0536, *p* < 0.001), which was consistent with Figure 2.

## 5 Discussion

This study aimed to examine the relationship between internet use and depression in older adults within the context of aging and digitization. It also examined the mediating role of older adults’ volunteer activity participation in the community and their friend networks. The study’s findings were as follows:

First, we found that Internet use was linked to lower depression in older adults, and the mechanism was their friend networks. This is consistent with previous studies (34, 35). However, unlike previous research that explains this mechanism by focusing on social support (10), we aim to clarify it based on the socioemotional selectivity theory. This theory indicates that people tend to seek out pleasant experiences as they age. Compared to acquaintances, older adults have more positive emotional experiences with close social partners like family and friends (58). Moreover, older adults can terminate unsatisfying friendships within their social networks due to the voluntary nature of friendships (59). Therefore, the Internet can help older adults actively adjust their friend networks without being limited by time and space, thus keeping more intimate relationships, obtaining more emotional experiences, and decreasing depression levels. The study’s results also verify it, indicating that internet use is linked to an increase in the friend networks of older adults, and older adults’ friend network is related to a decrease in depression levels.

Second, our research discovered that engagement in voluntary activities mediated Internet usage and depression in older adults. This also aligns with previous research (11). Previous studies have pointed out the Internet’s role in breaking down communication barriers with volunteers (40) and providing information about voluntary activities (60), which encourages volunteer participation. Our study further focused on the intrinsic motivation for older adults’ volunteer participation, with older adults themselves at the core of our analysis. Older adults tend to choose goals that are more emotion-related (61). Volunteering helps individuals feel like they matter and are making a contribution (37, 38), which is essential for older adults to gain emotional fulfillment. Therefore, the Internet facilitates older adults’ engagement in volunteer activities to realize emotion-related goals. Our study’s results also indicate that Internet use is associated with an increase in voluntary activity participation, which is linked to a decrease in depression levels.

Third, we explored the chain mediating role of volunteer activity participation and friend network. There has been limited research focusing on both simultaneously (12). We mainly analyzed the chain mediation relationship in terms of voluntary motive. Older adults tend to maintain existing friendships and participate in volunteering to act on important values (51, 52) due to the relative importance of volunteering and staying with family and close friends (50). They gradually prefer to maintain existing friendships through volunteering rather than cultivate new friendships (18). From the perspective of voluntary motive, older adults engaging in volunteer work may not benefit their friend networks. In addition, older adults who volunteer may experience conflicts in their roles, such as caring for sick or disabled adults (53, 54). Moreover, older adults may be less likely to engage in volunteer activities with their close friends, especially in informal community volunteering. Therefore, we hypothesized that changes in voluntary motive and role conflicts could be connected to the diminishing of older adults’ friend networks. Our analysis confirms this hypothesis, indicating that volunteer activity participation and participation intensity are linked to a decrease in older adults’ friend networks. The empirical analysis results also validate the mechanism behind the relationship between internet usage and depression in older adults as a chain mediation.

This study inevitably has some limitations. Firstly, due to the original data constraints, the friend network was measured by the Lubben Social Network Scale. In future studies, it would be beneficial to divide friendships into ordinary and close friends to explore the heterogeneity of friend networks with different psychological distances. Secondly, this study did not examine whether volunteer activity participation in other places is related to depression in older adults. Further studies could expand the research scope. Last but not least, future studies may use longitudinal data or experimental designs to make causal inferences.

The study’s findings point toward some potential policy implications that could be considered: First, the internet allows older adults to maintain intimate relationships, prevent social bond deterioration, and avert depression. Hence, we suggest that the government should prioritize digital empowerment for older adults. The community could mobilize tech-savvy older adults to help other seniors adopt digital technology and encourage them to use the Internet. Second, older adults prioritize positive emotional experiences, and they can achieve this through participating in voluntary activities within the community, which can help reduce levels of depression. Considering time and space constraints, communities could be an important place for older adults to volunteer. Therefore, we suggest that the government could assist in creating older adult-friendly communities by establishing activity centers and recording the preferences of older adults. Social workers could collaborate with tech-savvy older adults to organize various voluntary activities, encouraging more senior citizens to participate and achieve a balance between online and offline activities in their lives. Third, due to changes in volunteer motivations and potential role conflicts, older adults experience a decrease in their friend networks during volunteering. Thus, we suggest that the community should consider the form of activities, such as online-based activities, that can be shared across communities or even regions, allowing older adults to engage with their friends and meet like-minded individuals. The community should also pay attention to the format of offline activities to help older adults make friends first rather than solely focusing on voluntary activities.

## 6 Conclusion

In summary, this study used various methods to test whether Internet use was associated with older adults’ depression. After conducting empirical research, we found a significant negative correlation between the two variables, which remained consistent across several robustness checks. We also found that volunteer activity participation and friend networks played a chain mediating role. Further analysis revealed that participation intensity and friend network played the same role in this relationship. We propose some recommendations based on the findings of our study, including mobilizing tech-savvy older adults to help others adopt digital technology and encouraging them to use the Internet. We also suggest that government could assist in creating older adult-friendly communities, and social workers could collaborate with tech-savvy older adults to organize various voluntary activities. In addition, we recommend that the community should consider the form of activities to help older adults make friends first, rather than solely focusing on voluntary activities.

## Data Availability

The original contributions presented in the study are included in the article/supplementary material, further inquiries can be directed to the corresponding author.
